# Lung-protective ventilation strategy in acute respiratory distress syndrome: a critical reappraisal of current practice

**DOI:** 10.1186/s13054-025-05675-2

**Published:** 2025-10-21

**Authors:** Kwang Joo Park

**Affiliations:** Department of Pulmonary and Critical Care Medicine, Ajou University School of Medicine, 164 World cup-ro, Suwon, Gyeonggi-do 16499 South Korea

**Keywords:** Acute respiratory distress syndrome, Lung-protective strategy, Low tidal volume ventilation, Ventilator-induced lung injury, Individualized strategy

## Abstract

Recognition of ventilator-induced lung injury has led to the development of lung-protective ventilation strategies, significantly influencing the management of acute respiratory distress syndrome (ARDS). By the end of the 20th century, five randomized controlled trials had compared the survival benefits of low tidal volume (VT) ventilation with those of traditional high VT ventilation. Two studies demonstrated favourable outcomes, most notably the landmark ARDS Network trial, which established the widely recommended VT of 6 mL/kg predicted body weight. However, the universal application of a fixed VT has been controversial, with poor adherence in clinical practice. The two trials used a greater contrast in VTs (6 vs. 12 mL/kg) than did the others (7–11 mL/kg) and incorporated methodological extremes, including toleration of elevated airway pressures or encouragement of unnecessary increases. In addition, disparities in underlying aetiologies and ventilatory parameters, such as unbalanced positive end-expiratory pressure and respiratory rates, may have influenced the results. There is no conclusive evidence to support the superiority of 6 mL/kg over intermediate VTs (7–10 mL/kg). Many subsequent studies have suggested that VT requirements should be individualized on the basis of lung mechanics and physiological status. The benefits of the current recommendations may be limited by factors such as the severity of hypoxemia, lung compliance, dead-space fraction, and inaccuracies in formula-based lung volume estimation. The goal of mechanical ventilation in ARDS patients is supportive rather than curative; therefore, a moderate approach is recommended in clinical practice. Further studies are needed to establish an individualized, patient-centred approach that allows more flexible and moderate settings.

## Background

Acute respiratory distress syndrome (ARDS) is associated with high mortality, although outcomes have improved with advancements in ventilatory strategies and overall supportive care [1]. Since its initial clinical description by Ashbaugh and Petty in 1967, mechanical ventilation (MV) has remained the cornerstone of ARDS management [2, 3]. Initially, high tidal volumes (VTs) of 12–15 mL/kg were commonly used to compensate for the loss of functional lung units due to atelectasis and alveolar oedema [4]. However, the concept of ventilator-induced lung injury (VILI) soon emerged and was supported by animal studies and clinical evidence in humans [5–7]. These insights prompted a paradigm shift towards lung-protective ventilation, emphasizing the limitation of VT and airway pressure to mitigate iatrogenic injury [8].

Towards the end of the 20th century, five randomized controlled trials (RCTs) were conducted to compare traditional high-VT ventilation directly with lung-protective low-VT strategies and evaluate the prognostic implications (Table 1) [9–13]. Of these, two studies demonstrated a survival benefit, and the pivotal ARDS Network (ARDSNet) trial led to the recommendation of a VT of 6 mL/kg predicted body weight (PBW) as the standard for ARDS management [13–15]. Furthermore, low-VT ventilation has been increasingly applied in patients without ARDS [16–18]. Efforts have been made to explore even lower VTs (< 6 mL/kg PBW) in the management of ARDS [19–21].

**Table 1:** Comparison of the five randomized controlled trials that evaluated the survival benefit of low-tidal-volume ventilation versus traditional high-tidal-volume ventilation

Studies (first author)	Enrolment dates	Publication date	Sub-groups	No. subjects		Body weight measure applied	Tidal volume (ml/kg)			Resp. rate (/min)		
				Total	Sub- group		Target	Actual, Day 1	Actual, Day 7	Rule for setting	Day 1	Day 7
Brochard, et al. [10]	Jan 1994 – Sep 1996	Jan 14, 1998	Low VT	116	58	DBW	6–10	7.1 ± 1.3	7.37 ± 1.3	None	–	–
			High VT		58		10–15	10.3 ± 1.7	10.7 ± 1.8	Adjusted to maintain PaCO_2_ at 38–42 mmHg	–	–
Stewart, et al. [12]	July 1995 – Sep 1996	Feb 5, 1998	Low VT	120	60	IBW	≤ 8	7.0 ± 0.7	6.8 ± 0.6	5–35/min, adjusted to maintain PaCO_2_ at 35–45 mmHg	22.1 ± 6.2	24.9 ± 6.5
			High VT		60		10–15	10.7 ± 1.4	10.1 ± 1.4		15.6 ± 5.0	19.2 ± 4.7
Amato, et al. [9]	Dec 1990 – July 1995	Feb 5, 1998	Low VT	53	29	BW	≤ 6*			< 30/min, allowed PaCO_2_ limit: 80 mmHg	19.3†	21.4†
			High VT		24		12*			10–24/min, adjusted to maintain PaCO_2_ at 35–38 mmHg	15.9†	18.8†
Brower et al. [11]	May 2, 1994 – Mar 1, 1996	Aug 1999	Low VT	52	26	IBW‡	5–8	7.8§	7.3§‖	Adjusted to maintain PaCO_2_ at 30–45 mmHg	–	–
			High VT		26		10–12	10.2§	10.2§‖		–	–
ARDSNet [13]	Mar 1996 – Mar 1999	May 2000	Low VT	861	432	PBW‡	6 (4–8)	6.2 ± 0.9	6.5 ± 1.4	Adjusted to maintain pH at 7.3–7.45	29 ± 7	30 ± 7
			High VT		429		12	11.8 ± 0.8	11.4 ± 1.4		16 ± 6	20 ± 7

Studies (first author)	Subgroups	PEEP (cmH_2_O)			Set pressure limit (cmH_2_O)	Pplat (cmH_2_O)		Primary outcome of interest	Survival analysis	Administration of Na bicarbonate
		Rule for setting	Day 1	Day 7		Day 1	Day 7			
Brochard, et al. [10]	Low VT	Increments of 5 mH_2_O (0–15) for the greatest improvement in oxygenation or the first level allowing PaO_2_/FIO_2_ >200 mmHg	10.7 ± 2.9	9.6 ± 3.0	Pplat ≤ 25-30	25.7 ± 5.0	24.5 ± 5.7	60-d mortality	NS	If pH < 7.05
	High VT		10.7 ± 2.3	8.5 ± 2.8	Ppeak ≤ 60	31.7 ± 6.6	30.5 ± 9.4			
Stewart, et al. [12]	Low VT	5–20 cmH_2_O, increments of 2.5 cmH_2_O to maintain the FiO2 ≤ 0.5, SaO2 = 89–93%	8.6 ± 3.0	9.6 ± 3.9	Ppeak ≤ 30	22.3 ± 5.4	20.0 ± 4.7	In-hospital mortality	NS	If pH ≤ 7.0, 2 mmol/kg every four h (up to three doses)
	High VT		7.2 ± 3.3	8.0 ± 3.6	Ppeak ≤ 50	26.8 ± 6.7	28.6 ± 7.2			
Amato, et al. [9]	Low VT	2 cmH_2_O higher than Pflex	16.3 ± 0.7	13.2 ± 0.4¶	Pdriv < 20, Ppeak < 40	31.8 ± 1.4	23.9 ± 0.7¶	28-d mortality	Significant	If pH < 7.2, 50 mmol/h
	High VT		6.9 ± 0.8	9.3 ± 0.5¶	None	34.4 ± 1.9	37.8 ± 1.2¶			
Brower, et al. [11]	Low VT	Specific protocol combinations of PEEP and FIO_2_	9.5§	6.8§‖	Pplat ≤ 30	26.5§	23§‖	In-hospital mortality	NS	If pH was < 7.30, permissible; if pH was < 7.20, ≥ 10 mEq/h
	High VT		8.2§	6.0§‖	Pplat ≤ 45–55	30.5§	29§‖			
ARDSNet [13]	Low VT	Specific protocol combinations of PEEP and FIO_2_	9.4 ± 3.6	8.1 ± 3.4	25 ≤ Pplat ≤ 30	25 ± 7	26 ± 7	In-hospital mortality	Significant	Applied, but not described
	High VT		8.6 ± 3.6	9.1 ± 4.2	45 ≤ Pplat ≤ 50	33 ± 9	37 ± 9			

Data are presented as numbers (n) and means ± SEMs (Amato and Brower studies) or means ± SDs (the other three studies). ARDS, acute respiratory distress syndrome; ARDSNet, ARDS Network; VT, Tidal volume; Low VT, low-tidal-volume ventilation group; High VT, high-tidal-volume ventilation group; DBW, dry body weight; IBW, ideal body weight; BW, body weight; PBW, predicted body weight; Resp. rate, respiratory rate; PEEP, positive end-expiratory pressure; Pplat, plateau airway pressure; Ppeak, peak airway pressure; Pdriv, driving pressure (Pplat – PEEP); NS, not significant; Pflex, the lower inflection point on the inspiratory pressure–volume curve; Na bicarbonate, sodium bicarbonate

* These levels were strictly maintained with minimal variations according to the study design, although actual tidal volumes cannot be presented without body weight data; †estimated from tidal volume and minute volume data; ‡Devine’s formula; §visually estimated values from the figures; ‖values on Day 5; ¶ mean values on Days 2–7

Adherence to the recommended VT of 6 mL/kg PBW has been consistently low, at 20–39%, with actual clinical practice often involving higher VTs [22–24]. This suboptimal compliance has been attributed to multiple factors, including challenges in calculating and applying PBW accurately, limited clinician awareness or acceptance of permissive hypercapnia, concerns regarding increased sedation requirements, and the risk of hypoventilation and refractory hypoxemia [25–27]. Notably, deviations from guideline-recommended volumes should not always be interpreted as disregard for protocols. In many cases, clinicians may determine that the standardized VT is inappropriate or inadequate for a given patient’s physiological condition and may elect to increase it for safety and individualized care considerations.

## Methods

The primary candidates for this review were clinical studies that assessed outcome parameters on the basis of the VT settings used in the MV of patients with ARDS.

The five aforementioned RCTs are key articles in this review. Although those studies are somewhat outdated, having been published between 1998 and 2000, they continue to exert the most significant influence on current ventilatory care in ARDS management because no comparable studies have been conducted since 2000 [9–13] (Table 1).

Two uncontrolled, retrospective real-world analyses were also selected for this review because they provided comprehensive evaluations involving relatively large numbers of patients [26, 28].

Additionally, two articles that addressed the five RCTs were selected. In one study, a meta-analysis of the data was conducted [29]; in the other study, a secondary analysis was performed in which the original data was used as the basis for assessing the implications of the physiological and mechanical ventilatory parameters [30].

In addition to these primary articles, various relevant clinical research and review papers were selected and analysed.

## Integrated results and discussion

### Reassessment of landmark studies on mechanical ventilation in ARDS

To evaluate the efficacy of lung-protective low-VT ventilation compared to conventional high-VT ventilation, five RCTs have been conducted using comparable methodologies. Of these, three studies reported no significant difference in survival outcomes, and two studies demonstrated a survival benefit with the low-VT strategy. Notably, the ARDSNet trial received the greatest recognition and established the foundation for current MV standards; its influence stems from its rigorous design as a well-controlled, multicentre study with superior statistical power and a relatively large patient population [13].

Concerns have been raised regarding the fixed application of low-VT ventilation, as the benefit thereof may vary according to mechanical parameters such as the driving pressure. It can also lead to air hunger and double triggering, requiring excessive sedation, and may be detrimental in patients with severe hypoventilation and acidosis or obstructive lung disease [31–35]. Several studies have suggested that the benefits of low-VT ventilation may be limited in severe ARDS patients and that its effectiveness can vary depending on the elastance or compliance of the respiratory system [30, 36]. Accordingly, it has been proposed that VT should be individualized on the basis of driving pressure and the severity of hypoxemia [37–39]. As there is no single, definitive method to quantify the ventilation requirements of any given individual, integrated observations of vital physiology remain the best guide for moment-by-moment lung protection at the bedside. VILI risk varies not only inversely with the size of the aerated lung but also with the nature, severity, and stage of acute injury. Single numerical values for ventilation parameters, such as VT, pressure and positive end-expiratory pressure (PEEP), are not relevant for all individuals [8, 40, 41].

There is no basis to regard the recommended VT of 6 mL/kg PBW as optimal; this volume was directly compared only to 12 mL/kg, without evaluation against intermediate volumes of 7–11 mL/kg [33, 34]. The ARDSNet trial addressed this concern by comparing its results with those of three trials that did not demonstrate a survival benefit. In the ARDSNet study, the low-VT group received approximately 6 mL/kg PBW, whereas in the studies that did not show a benefit, the low VTs were closer to 7 mL/kg. Notably, the high-VT arms in both the ARDSNet and other trials were comparable, averaging approximately 12 mL/kg PBW when adjusted using the PBW. On the basis of these comparisons, a possible inference was drawn: since 6 mL/kg yielded better outcomes than 12 mL/kg in the ARDSNet trial did, and 12 mL/kg resulted in outcomes similar to those of 7 mL/kg in the studies that did not show a benefit, 6 mL/kg was hypothesized to also be superior to the range of higher VTs of 7–12 mL/kg. However, this conclusion remains inferential because direct comparisons with intermediate volumes have not been systematically tested.

Several concerns have been raised regarding the interpretation of the findings of the studies that reported a survival benefit. First, discrepancies in the adjustment of body weight across studies complicate direct comparisons. Of the three trials that did not show a benefit, two used ideal body weight (IBW), and one used dry body weight [10–12]. Although these methods are conceptually similar and are intended to reflect lean body mass and lung size, they were selected on the basis of prevailing trends or individual preferences. Furthermore, all weight estimates were calculated indirectly using height and sex rather than being obtained through direct measurement. In the ARDSNet trial, the PBW was calculated using the Devine formula, which is also widely used to determine IBW [11, 42]. Notably, IBW and PBW yield very similar values, regardless of the specific formula applied [43]. Dry body weight is typically estimated using formulas such as the Devine equation because accurate measurement is challenging even when advanced technologies are used [44]. Thus, converting estimated body weights across studies offers limited value and may introduce unnecessary inconsistencies, and analysing the data using the originally reported values without adjustment may be more appropriate. The initial VTs in the ARDSNet trial were approximately 6.2 and 11.8 ml/kg, whereas in the three studies that did not show a benefit, they ranged from approximately 7–10.7 ml/kg (rather than 7–12 ml/kg, as previously stated) (Table 1). Thus, the VT ranges in the ARDSNet study did not overlap with those in the trials that did not show a benefit, thereby precluding direct and indirect comparisons between 6 mL/kg and higher VTs of 7–11 mL/kg. As a result, although the ARDSNet trial primarily demonstrated the harm of excessively high VTs, it did not definitively establish 6 mL/kg PBW as the optimal target. To date, no RCT has directly compared the ARDSNet-recommended low VT (6 mL/kg PBW) with intermediate volumes (7–10 ml/kg PBW). However, one multicentre retrospective study revealed no significant difference in outcomes between groups receiving mean VTs of 6.7 ml/kg and 11.2 ml/kg PBW [28]. Similarly, a retrospective review of 111 real-world ARDS patients revealed that a mean VT of 9.5 ml/kg PBW was not inferior to that of 6.1 ml/kg PBW in terms of 28-day or 1-year mortality [26].

Second, the two studies that demonstrated a survival benefit employed fixed VTs of 6 and 12 mL/kg PBW for the intervention and control groups, respectively. In contrast, the three studies that did not show a benefit adopted a more flexible approach, setting VTs within the 7–11 ml/kg range and adjusting them based on airway pressure parameters such as peak airway pressure (Ppeak) and plateau airway pressure (Pplat) (Table 1). Notably, in the ARDSNet trial, a lower limit Pplat threshold of 45 cmH₂O was established in the high-VT group, below which the VT was titrated up to 12 mL/kg [13]. Another study applied a similarly rigid protocol, enforcing fixed VTs without incorporating a pressure safety margin in the high-VT arm. This approach resulted in substantial differences in Pplat between groups, despite the small sample size [9]. Such protocols may not have reflected the prevailing standards of care at the time, particularly because the risks of VILI had been recognized for more than a decade [5–7, 45–48]. These rigid and arguably excessive methodologies may have contributed to the poorer outcomes observed in the high-VT groups of the two studies showing a benefit. A meta-analysis of the above five RCTs revealed that Pplat values in the high-VT groups were significantly higher in studies that showed a benefit than those that did not but did not specifically address the methodological issues [29]. The unregulated application of high VTs, irrespective of these pressure thresholds, may have led to exaggerated elevations in Pplat, contributing to the observed outcome disparities.

Third, respiratory rates were set unusually high in the low-VT groups to compensate for reduced minute ventilation, whereas PEEP levels were lower in the high-VT groups [9, 13]. These discrepancies in ventilator parameters introduce significant confounding factors that undermine the fairness of the comparison and deviate from reasonable ventilation practices.

Another potential contributor to the outcome differences in the ARDSNet trial lies in the distribution of the underlying aetiologies of ARDS. Notably, trauma-related ARDS is associated with more favourable outcomes than ARDS due to pulmonary causes such as pneumonia [49, 50]. A simple chi-square estimation based on published data revealed that the number of pneumonia cases was slightly greater and that the number of trauma cases was significantly lower in the high-VT group than in the low-VT group [13].

The ARDSNet trial suggested that the use of parenteral bicarbonate to mitigate acidosis may have influenced outcomes. However, this rationale is unconvincing because all three studies that did not show a benefit explicitly reported the administration of sodium bicarbonate for acid‒base management [10–13] (Table 1).

### Further considerations when low-tidal-volume ventilation is applied

The normal VT in mammals, including humans, is approximately 6.3 mL/kg IBW at rest [51]. Considering variation and changing demands, the physiological VT in healthy individuals is typically 6–8 mL/kg [13, 52]. In ARDS, however, the number of functioning lung units is significantly reduced because of alveolar collapse, oedema, or consolidation. To minimize regional overdistension and VILI, the VT must be proportionally decreased to match the diminished aerated lung volume [53, 54]. However, volume- and pressure-induced injuries are not the only factors that must be considered when ventilatory parameters are set in ARDS. The primary objective of MV is to maintain adequate gas exchange and avoid further damage to the lungs. In ARDS, the physiological dead-space fraction can rise dramatically—often exceeding 0.5–0.6 compared with normal values < 0.3—and this increase is independently associated with greater mortality [55–58]. In patients with a significantly elevated dead space fraction, the application of a low-VT strategy may result in significant hypoventilation and severe hypoxemia. To compensate for this impaired gas exchange, clinicians are often compelled to use excessively high respiratory rates and elevated PEEP levels. These compensatory measures carry potential drawbacks, such as intrinsic PEEP, and may contribute to the lack of consistent survival benefits associated with low-VT ventilation, particularly in patients with high respiratory system elastance or profound hypoxia [30, 39].

Beyond these physiological concerns, the use of formula-based estimations to determine VT introduces additional limitations. VT may be inappropriately set for individual patients because of inaccuracies in height measurement, estimation errors, and interindividual variability in lung capacity [59, 60]. In critically ill patients, even a small degree of underestimation can have serious or catastrophic consequences. When life-support parameters, such as VT, are determined on the basis of crude estimations rather than direct physiological assessment, overly rigid adherence to calculated values—without allowance for individual variation—may be detrimental.

The result of the five RCTs discussed above show that the actual VTs administered to patients in the low-VT groups consistently exceeded the protocol-defined targets, including in the ARDSNet trial. This observation implies that the prescribed VTs may have been perceived as insufficient by the treating physicians, who likely adjusted them upwards in response to clinical needs, such as ensuring adequate ventilation or addressing safety concerns.

VILI arises from mechanical stress and biologically mediated processes, collectively referred to as “biotrauma” [61]. MV triggers the release of inflammatory mediators, such as cytokines [62, 63]. This cytokine surge has been associated with the development of multiorgan failure, a key determinant of poor outcomes in ARDS, although definitive evidence that inflammatory mediators are direct causative agents of multiorgan dysfunction remains lacking [64, 65]. These mediators may represent epiphenomena, which are byproducts of the disease process rather than primary drivers of pathology. Moreover, inflammatory cytokines play essential roles in host defence, tissue repair, and immune regulation [66, 67].

### Back to basics and lessons from the past

The primary function of MV is to provide supportive care rather than to serve as a definitive therapeutic intervention. A prudent and restrained approach is warranted when advanced respiratory support modalities are applied. Although numerous adjunctive strategies for MV have been investigated, their clinical outcomes have generally fallen short of initial expectations, and many are not routinely used in contemporary critical care practice. In particular, inhaled nitric oxide, which was once widely used, is no longer recommended, nor is high-frequency ventilation [68, 69].

Although the concepts of VILI and biotrauma from excessive ventilation are well known and have significantly advanced the field of critical care medicine, paradoxically, overly aggressive ventilatory strategies may lead to further harm.

Dr. David G. Ashbaugh, who first described ARDS, passed away in 2022. A surgeon by training, Dr. Ashbaugh was not extensively involved in critical care research. However, he remains one of the most influential pioneers in the field of critical-care medicine, having saved more lives than many of the most celebrated figures have. His discovery of the profound and immediate benefits of PEEP occurred somewhat serendipitously during a desperate attempt to save a young patient’s life. The clinical improvement was so dramatic and unequivocal that conducting further trials to assess the necessity of PEEP was deemed unnecessary and ethically unjustifiable. Dr. Ashbaugh, together with the late Dr. Thomas L. Petty and colleagues, first reported cases of ARDS in *The Lancet* after their manuscript had been rejected by leading American journals. Notably, one of the main reasons for rejection was the inclusion of PEEP, which at the time was considered potentially harmful during MV. This historical episode highlights how seemingly rational judgements, grounded in the prevailing knowledge of the time, may later be recognized as clear misjudgements. Similarly, the current emphasis on low-VT ventilation may, in retrospect, be viewed as a misplaced endeavour. As previously discussed, life-sustaining interventions should not be based on overly rigid application of low-VT settings derived from crude conversion formulas without careful consideration of the physiological status of the patient and the mechanical parameters of the respiratory system. Low-VT ventilation, although beneficial in many cases, may function as a double-edged sword: further reduction in VT in pursuit of uncertain and marginal gains may pose significant risks, particularly in the absence of precise and individualized methods for determining optimal ventilatory requirements.

## Conclusion

Lung protection strategies, including low-VT ventilation in ARDS patients, represent among the most significant advancements in critical care medicine. However, there is no definitive evidence to support the superiority of a fixed VT of 6 mL/kg PBW or lower over intermediate VTs. Both excessively high and overly low VTs may be harmful in patients with ARDS, particularly in the context of severe hypoxia, markedly reduced pulmonary compliance, or a high dead-space fraction. To ensure safe and effective MV, existing protocols should be critically re-evaluated in light of emerging evidence. A therapeutic approach grounded in moderation, individualized assessment, and clinical prudence is essential for the optimal management of ARDS.

## Data Availability

No datasets were generated or analysed during the current study.

## Abbreviations

ARDS: Acute respiratory distress syndrome
MV: Mechanical ventilation
VT: Tidal volume
VILI: Ventilator-induced lung injury
RCT: Randomized controlled trial
ARDSNet: ARDS Network
PBW: Predicted body weight
PEEP: Positive end-expiratory pressure
IBW: Ideal body weight
Ppeak: Peak airway pressure
Pplat: Plateau airway pressure
Pdriv: Driving pressure

## References

[1] Meyer NJ, Gattinoni L, Calfee CS. Acute respiratory distress syndrome. Lancet. 2021;398(10300):622–37. 34217425 10.1016/S0140-6736(21)00439-6 PMC8248927

[2] Petty TL, Ashbaugh DG. The adult respiratory distress syndrome. Clinical features, factors influencing prognosis and principles of management. Chest. 1971;60(3):233–9. 4937358 10.1378/chest.60.3.233

[3] Ashbaugh DG, Petty TL, Bigelow DB, Harris TM. Continuous positive-pressure breathing (CPPB) in adult respiratory distress syndrome. J Thorac Cardiovasc Surg. 1969;57(1):31–41. 4883575

[4] Kollef MH, Schuster DP. The acute respiratory distress syndrome. N Engl J Med. 1995;332(1):27–37. 7646623 10.1056/NEJM199501053320106

[5] Webb HH, Tierney DF. Experimental pulmonary edema due to intermittent positive pressure ventilation with high inflation pressures. Protection by positive end-expiratory pressure. Am Rev Respir Dis. 1974;110(5):556–65. 4611290 10.1164/arrd.1974.110.5.556

[6] John E, Ermocilla R, Golden J, McDevitt M, Cassady G. Effects of intermittent positive-pressure ventilation on lungs of normal rabbits. Br J Exp Pathol. 1980;61(3):315–23. 6775666 PMC2041578

[7] Kolobow T, Moretti MP, Fumagalli R, Mascheroni D, Prato P, Chen V, et al. Severe impairment in lung function induced by high peak airway pressure during mechanical ventilation. An experimental study. Am Rev Respir Dis. 1987;135(2):312–5. 3544984 10.1164/arrd.1987.135.2.312

[8] Whitehead T, Slutsky AS. The pulmonary physician in critical care * 7: ventilator induced lung injury. Thorax. 2002;57(7):635–42. 12096209 10.1136/thorax.57.7.635 PMC1746372

[9] Amato MB, Barbas CS, Medeiros DM, Magaldi RB, Schettino GP, Lorenzi-Filho G, et al. Effect of a protective-ventilation strategy on mortality in the acute respiratory distress syndrome. N Engl J Med. 1998;338(6):347–54. 9449727 10.1056/NEJM199802053380602

[10] Brochard L, Roudot-Thoraval F, Roupie E, Delclaux C, Chastre J, Fernandez-Mondejar E, et al. Tidal volume reduction for prevention of ventilator-induced lung injury in acute respiratory distress syndrome. The multicenter trail group on tidal volume reduction in ARDS. Am J Respir Crit Care Med. 1998;158(6):1831–8. 9847275 10.1164/ajrccm.158.6.9801044

[11] Brower RG, Shanholtz CB, Fessler HE, Shade DM, White P Jr., Wiener CM, et al. Prospective, randomized, controlled clinical trial comparing traditional versus reduced tidal volume ventilation in acute respiratory distress syndrome patients. Crit Care Med. 1999;27(8):1492–8. 10470755 10.1097/00003246-199908000-00015

[12] Stewart TE, Meade MO, Cook DJ, Granton JT, Hodder RV, Lapinsky SE, et al. Evaluation of a ventilation strategy to prevent barotrauma in patients at high risk for acute respiratory distress syndrome. Pressure- and Volume-Limited ventilation strategy group. N Engl J Med. 1998;338(6):355–61. 9449728 10.1056/NEJM199802053380603

[13] Acute Respiratory Distress, Syndrome N, Brower RG, Matthay MA, Morris A, Schoenfeld D, Thompson BT, et al. Ventilation with lower tidal volumes as compared with traditional tidal volumes for acute lung injury and the acute respiratory distress syndrome. N Engl J Med. 2000;342(18):1301–8. 10793162 10.1056/NEJM200005043421801

[14] Suter PM, Fairley B, Isenberg MD. Optimum end-expiratory airway pressure in patients with acute pulmonary failure. N Engl J Med. 1975;292(6):284–9. 234174 10.1056/NEJM197502062920604

[15] Fan E, Del Sorbo L, Goligher EC, Hodgson CL, Munshi L, Walkey AJ, et al. An official American thoracic society/European society of intensive care medicine/Society of critical care medicine clinical practice guideline: mechanical ventilation in adult patients with acute respiratory distress syndrome. Am J Respir Crit Care Med. 2017;195(9):1253–63. 28459336 10.1164/rccm.201703-0548ST

[16] Lipes J, Bojmehrani A, Lellouche F. Low tidal volume ventilation in patients without acute respiratory distress syndrome: A paradigm shift in mechanical ventilation. Crit Care Res Pract. 2012;2012:416862. 22536499 10.1155/2012/416862 PMC3318889

[17] Futier E, Constantin JM, Paugam-Burtz C, Pascal J, Eurin M, Neuschwander A, et al. A trial of intraoperative low-tidal-volume ventilation in abdominal surgery. N Engl J Med. 2013;369(5):428–37. 23902482 10.1056/NEJMoa1301082

[18] Neto AS, Simonis FD, Barbas CS, Biehl M, Determann RM, Elmer J, et al. Lung-Protective ventilation with low tidal volumes and the occurrence of pulmonary complications in patients without acute respiratory distress syndrome: A systematic review and individual patient data analysis. Crit Care Med. 2015;43(10):2155–63. 26181219 10.1097/CCM.0000000000001189

[19] Richard JC, Marque S, Gros A, Muller M, Prat G, Beduneau G, et al. Feasibility and safety of ultra-low tidal volume ventilation without extracorporeal circulation in moderately severe and severe ARDS patients. Intensive Care Med. 2019;45(11):1590–8. 31549225 10.1007/s00134-019-05776-x

[20] Costa EL, Amato MB. Ultra-protective tidal volume: how low should we go? Crit Care. 2013;17(2):127. 23551995 10.1186/cc12556 PMC3672527

[21] Richard JC, Terzi N, Yonis H, Chorfa F, Wallet F, Dupuis C, et al. Ultra-low tidal volume ventilation for COVID-19-related ARDS in France (VT4COVID): a multicentre, open-label, parallel-group, randomised trial. Lancet Respir Med. 2023;11(11):991–1002. 37453445 10.1016/S2213-2600(23)00221-7

[22] Samanta RJ, Ercole A, Harris S, Summers C. Low tidal volume ventilation is poorly implemented for patients in North American and united Kingdom ICUs using electronic health records. Chest. 2024;165(2):333–47. 37775039 10.1016/j.chest.2023.09.021 PMC10851261

[23] Poole J, McDowell C, Lall R, Perkins G, McAuley D, Gao F, et al. Individual patient data analysis of tidal volumes used in three large randomized control trials involving patients with acute respiratory distress syndrome. Br J Anaesth. 2017;118(4):570–5. 28403395 10.1093/bja/aew465 PMC8542892

[24] Wilkins D, Lane AS, Orde SR. Audit of low tidal volume ventilation in patients with hypoxic respiratory failure in a tertiary Australian intensive care unit. Anaesth Intensive Care. 2021;49(4):301–8. 34324389 10.1177/0310057X21993132

[25] Nadeem RN, Elhoufi AM, Soliman MA, Bon I, Obaida ZA, Hussien MM, et al. Clinical predictors of adherence to low tidal volume ventilation practice: is it different on weekend and night shifts? Cureus. 2019;11(6):e4844. 31410327 10.7759/cureus.4844 PMC6684108

[26] Chen YF, Lim CK, Ruan SY, Jerng JS, Lin JW, Kuo PH, et al. Factors associated with adherence to low-tidal volume strategy for acute lung injury and acute respiratory distress syndrome and their impacts on outcomes: an observational study and propensity analysis. Minerva Anestesiol. 2014;80(11):1158–68. 24569355

[27] Midega TD, Bozza FA, Machado FR, Guimaraes HP, Salluh JI, Nassar AP Jr., et al. Organizational factors associated with adherence to low tidal volume ventilation: a secondary analysis of the CHECKLIST-ICU database. Ann Intensive Care. 2020;10(1):68. 32488524 10.1186/s13613-020-00687-3 PMC7266115

[28] Wu SH, Kor CT, Li CY, Hsiao YC. Intermediate tidal volume is an acceptable option for ventilated patients with acute respiratory distress syndrome. Med Intensiva (Engl Ed). 2022;46(11):609–18. 36344012 10.1016/j.medine.2022.03.002 PMC9633924

[29] Eichacker PQ, Gerstenberger EP, Banks SM, Cui X, Natanson C. Meta-analysis of acute lung injury and acute respiratory distress syndrome trials testing low tidal volumes. Am J Respir Crit Care Med. 2002;166(11):1510–4. 12406836 10.1164/rccm.200208-956OC

[30] Goligher EC, Costa ELV, Yarnell CJ, Brochard LJ, Stewart TE, Tomlinson G, et al. Effect of Lowering Vt on mortality in acute respiratory distress syndrome varies with respiratory system elastance. Am J Respir Crit Care Med. 2021;203(11):1378–85. 33439781 10.1164/rccm.202009-3536OC

[31] Lee PC, Helsmoortel CM, Cohn SM, Fink MP. Are low tidal volumes safe? Chest. 1990;97(2):430–4. 2288551 10.1378/chest.97.2.430

[32] Wright BJ, Slesinger TL. Low tidal volume should not routinely be used for emergency department patients requiring mechanical ventilation. Ann Emerg Med. 2012;60(2):216–7. 22818370 10.1016/j.annemergmed.2011.08.006 PMC9946161

[33] Tobin MJ. The dethroning of 6 ml/kg as the Go-To setting in acute respiratory distress syndrome. Am J Respir Crit Care Med. 2021;204(7):868–9. 34186011 10.1164/rccm.202105-1320LE PMC8528533

[34] Pellegrini M, Del Sorbo L, Ranieri VM. Finding the optimal tidal volume in acute respiratory distress syndrome. Intensive Care Med. 2024;50(7):1154–6. 38740616 10.1007/s00134-024-07440-5 PMC11245429

[35] Tobin MJ. ARDS: hidden perils of an overburdened diagnosis. Crit Care. 2022;26(1):392. 36528765 10.1186/s13054-022-04271-y PMC9758457

[36] Deans KJ, Minneci PC, Cui X, Banks SM, Natanson C, Eichacker PQ. Mechanical ventilation in ARDS: one size does not fit all. Crit Care Med. 2005;33(5):1141–3. 15891350 10.1097/01.ccm.0000162384.71993.a3

[37] Urner M, Juni P, Hansen B, Wettstein MS, Ferguson ND, Fan E. Time-varying intensity of mechanical ventilation and mortality in patients with acute respiratory failure: a registry-based, prospective cohort study. Lancet Respir Med. 2020;8(9):905–13. 32735841 10.1016/S2213-2600(20)30325-8 PMC7906666

[38] Urner M, Juni P, Rojas-Saunero LP, Hansen B, Brochard LJ, Ferguson ND, et al. Limiting dynamic driving pressure in patients requiring mechanical ventilation. Crit Care Med. 2023;51(7):861–71. 36971437 10.1097/CCM.0000000000005844

[39] Shen Y, Cai G, Gong S, Dong L, Yan J, Cai W. Interaction between low tidal volume ventilation strategy and severity of acute respiratory distress syndrome: a retrospective cohort study. Crit Care. 2019;23(1):254. 31300012 10.1186/s13054-019-2530-6 PMC6626332

[40] Gattinoni L, Tonetti T, Quintel M. Regional physiology of ARDS. Crit Care. 2017;21(Suppl 3):312. 29297365 10.1186/s13054-017-1905-9 PMC5751536

[41] Merola R, Vargas M, Battaglini D Ventilator-Induced Lung Injury: The Unseen Challenge in Acute Respiratory Distress Syndrome Management. J Clin Med 2025;14(11):3910. 40507672 10.3390/jcm14113910 PMC12156453

[42] Pai MP, Paloucek FP. The origin of the ideal body weight equations. Ann Pharmacother. 2000;34(9):1066–9. 10981254 10.1345/aph.19381

[43] MacDonald JJ, Moore J, Davey V, Pickering S, Dunne T. The weight debate. J Intensive Care Soc. 2015;16(3):234–8. 28979416 10.1177/1751143714565059 PMC5606431

[44] Germain MJ, Greco BA, Hodgins S, Chapagain B, Thadhani R, Wojciechowski D, et al. Assessing accuracy of estimated dry weight in Dialysis patients post transplantation: the kidney knows best. J Nephrol. 2021;34(6):2093–7. 34031847 10.1007/s40620-021-01029-7 PMC8611116

[45] Dreyfuss D, Soler P, Basset G, Saumon G. High inflation pressure pulmonary edema. Respective effects of high airway pressure, high tidal volume, and positive end-expiratory pressure. Am Rev Respir Dis. 1988;137(5):1159–64. 3057957 10.1164/ajrccm/137.5.1159

[46] Dreyfuss D, Saumon G. Barotrauma is volutrauma, but which volume is the one responsible? Intensive Care Med. 1992;18(3):139–41. 1644960 10.1007/BF01709236

[47] Kumar A, Pontoppidan H, Falke KJ, Wilson RS, Laver MB. Pulmonary barotrauma during mechanical ventilation. Crit Care Med. 1973;1(4):181–6. 4587509 10.1097/00003246-197307000-00001

[48] Petersen GW, Baier H. Incidence of pulmonary barotrauma in a medical ICU. Crit Care Med. 1983;11(2):67–9. 6337021 10.1097/00003246-198302000-00002

[49] Steltzer H, Krafft P. Prognosis of ARDS patients: light at the end of the tunnel? Intensive Care Med. 1997;23(8):803–5. 9310795 10.1007/s001340050414

[50] Xie R, Tan D, Liu B, Xiao G, Gong F, Zhang Q et al. Acute respiratory distress syndrome (ARDS): from mechanistic insights to therapeutic strategies. MedComm (2020). 2025;6(2):e70074. 10.1002/mco2.70074 PMC11769712 39866839

[51] Villar J, Kacmarek RM, Hedenstierna G. From ventilator-induced lung injury to physician-induced lung injury: why the reluctance to use small tidal volumes? Acta Anaesthesiol Scand. 2004;48(3):267–71. 14982557 10.1111/j.0001-5172.2004.0340.x

[52] Bowton DL, Scott LK. Ventilatory management of the noninjured lung. Clin Chest Med. 2016;37(4):701–10. 27842750 10.1016/j.ccm.2016.07.010

[53] Gattinoni L, Pesenti A. The concept of baby lung. Intensive Care Med. 2005;31(6):776–84. 15812622 10.1007/s00134-005-2627-z

[54] Pfeilsticker F, Serpa Neto A. Lung-protective’ ventilation in acute respiratory distress syndrome: still a challenge? J Thorac Dis. 2017;9(8):2238–41. 28932514 10.21037/jtd.2017.06.145 PMC5594148

[55] Vender RL, Betancourt MF, Lehman EB, Harrell C, Galvan D, Frankenfield DC. Prediction equation to estimate dead space to tidal volume fraction correlates with mortality in critically ill patients. J Crit Care. 2014;29(2):e3171–3. 10.1016/j.jcrc.2013.12.009 24581935

[56] Ferluga M, Lucangelo U, Blanch L. Dead space in acute respiratory distress syndrome. Ann Transl Med. 2018;6(19):388. 30460262 10.21037/atm.2018.09.46 PMC6212367

[57] Nuckton TJ, Alonso JA, Kallet RH, Daniel BM, Pittet JF, Eisner MD, et al. Pulmonary dead-space fraction as a risk factor for death in the acute respiratory distress syndrome. N Engl J Med. 2002;346(17):1281–6. 11973365 10.1056/NEJMoa012835

[58] Doorduin J, Nollet JL, Vugts MP, Roesthuis LH, Akankan F, van der Hoeven JG, et al. Assessment of dead-space ventilation in patients with acute respiratory distress syndrome: a prospective observational study. Crit Care. 2016;20(1):121. 27145818 10.1186/s13054-016-1311-8 PMC4857382

[59] O’Brien ID, Shacklock E, Middleditch A, Bigham C. Inaccuracies in calculating predicted body weight and its impact on safe ventilator settings. J Intensive Care Soc. 2016;17(3):191–5. 28979490 10.1177/1751143715626163 PMC5606514

[60] Sasko B, Thiem U, Christ M, Trappe HJ, Ritter O, Pagonas N. Size matters: an observational study investigating estimated height as a reference size for calculating tidal volumes if low tidal volume ventilation is required. PLoS ONE. 2018;13(6):e0199917. 29958278 10.1371/journal.pone.0199917 PMC6025863

[61] Slutsky AS, Ranieri VM. Ventilator-induced lung injury. N Engl J Med. 2013;369(22):2126–36. 24283226 10.1056/NEJMra1208707

[62] Curley GF, Laffey JG, Zhang H, Slutsky AS. Biotrauma and Ventilator-Induced lung injury: clinical implications. Chest. 2016;150(5):1109–17. 27477213 10.1016/j.chest.2016.07.019

[63] dos Santos CC, Slutsky AS. Protective ventilation of patients with acute respiratory distress syndrome. Crit Care. 2004;8(3):145–7. 15153229 10.1186/cc2849 PMC468895

[64] Amado-Rodriguez L, Del Busto C, Lopez-Alonso I, Parra D, Mayordomo-Colunga J, Arias-Guillen M, et al. Biotrauma during ultra-low tidal volume ventilation and venoarterial extracorporeal membrane oxygenation in cardiogenic shock: a randomized crossover clinical trial. Ann Intensive Care. 2021;11(1):132. 34453620 10.1186/s13613-021-00919-0 PMC8397875

[65] Guervilly C, Fournier T, Chommeloux J, Arnaud L, Pinglis C, Baumstarck K, et al. Ultra-lung-protective ventilation and biotrauma in severe ARDS patients on veno-venous extracorporeal membrane oxygenation: a randomized controlled study. Crit Care. 2022;26(1):383. 36510324 10.1186/s13054-022-04272-x PMC9744058

[66] Gao M, Guo H, Dong X, Wang Z, Yang Z, Shang Q, et al. Regulation of inflammation during wound healing: the function of mesenchymal stem cells and strategies for therapeutic enhancement. Front Pharmacol. 2024;15:1345779. 38425646 10.3389/fphar.2024.1345779 PMC10901993

[67] Nirenjen S, Narayanan J, Tamilanban T, Subramaniyan V, Chitra V, Fuloria NK, et al. Exploring the contribution of pro-inflammatory cytokines to impaired wound healing in diabetes. Front Immunol. 2023;14:1216321. 37575261 10.3389/fimmu.2023.1216321 PMC10414543

[68] Grotberg JC, Reynolds D, Kraft BD. Management of severe acute respiratory distress syndrome: a primer. Crit Care. 2023;27(1):289. 37464381 10.1186/s13054-023-04572-w PMC10353255

[69] Qadir N, Chen JT. Adjunctive therapies in ARDS: the disconnect between clinical trials and clinical practice. Chest. 2020;157(6):1405–6. 32505301 10.1016/j.chest.2020.03.022 PMC7267806

