# Correction: Lung-protective ventilation strategy in acute respiratory distress syndrome: a critical reappraisal of current practice

**DOI:** 10.1186/s13054-026-06087-6

**Published:** 2026-05-29

**Authors:** Kwang Joo Park

**Affiliations:** Department of Pulmonary and Critical Care Medicine, Ajou University School of Medicine, 164 World cup-ro, Suwon, 16499 Gyeonggi-do South Korea

**Correction**: **Critical Care (2025) 29:444**

10.1186/s13054-025-05675-2

Following publication of the original article [1], the author identified errors in the ‘Back to basics and lessons from the past’ section. In the given section, the name of Dr. David George Ashbaugh was incorrectly presented as David O. Ashbaugh instead of David G. Ashbaugh. Additionally, his year of death was mistakenly stated as 2016, while he actually passed away in 2022. 

The sentence in the 'Integrated results and discussion' section at the sub-section 'Back to basics and lessons from the past' originally reads:

Dr. David O. Ashbaugh, who first described ARDS, passed away in 2016.

The sentence currently reads:

Dr. David G. Ashbaugh, who first described ARDS, passed away in 2022.

The author sincerely apologizes for these errors regarding such a dedicated and accomplished individual and extends his deepest condolences to his family.

The correct name and the year David G. Ashbaugh deceased are indicated in this correction article and the original article [1] has been corrected.
